# Shotgun EM of mycobacterial protein complexes during stationary phase stress

**DOI:** 10.1016/j.crstbi.2020.09.002

**Published:** 2020-09-22

**Authors:** Angela M. Kirykowicz, Jeremy D. Woodward

**Affiliations:** a Department of Biochemistry, University of Cambridge, Sanger Building, Tennis Court Road, Cambridge, CB2 1GA, UK; b Division of Medical Biochemistry and Structural Biology, Department of Integrative Biomedical Sciences, University of Cape Town, Anzio Road, Observatory, 7925, Cape Town, South Africa; c Structural Biology Research Unit, University of Cape Town, South Africa

**Keywords:** Three dimensional electron microscopy (3DEM), Protein structure, Mycobacteria, Oxidative stress, Structural proteomics, Shotgun approach

## Abstract

There is little structural information about the protein complexes conferring resistance in *Mycobacterium tuberculosis (Mtb)* to anti-microbial oxygen and nitrogen radicals in the phagolysosome. Here, we expose the model Mycobacterium, *Mycobacterium smegmatis,* to simulated oxidative-stress conditions and apply a shotgun EM method for the structural detection of the resulting protein assemblies. We identified: glutamine synthetase I, essential for *Mtb* virulence; bacterioferritin A, critical for *Mtb* iron regulation; aspartyl aminopeptidase M18, a protease; and encapsulin, which produces a cage-like structure to enclose cargo proteins. After further investigation, we found that encapsulin carries dye-decolourising peroxidase, a protein antioxidant, as its primary cargo under the conditions tested.

## Highlights

• Induce oxidative stress response in *Mycobacterium smegmatis*.
• Applied shotgun electron microscopy to find native protein complexes.
• Relion single particle reconstruction and identify by mass spectrometry.
• Found four protein complexes: encapsulin, GSI, BfrA, apeB.
• Encapsulin encloses dye-decolourising type peroxidase, an enzymatic antioxidant.

## Graphical abstract

**Figure:**
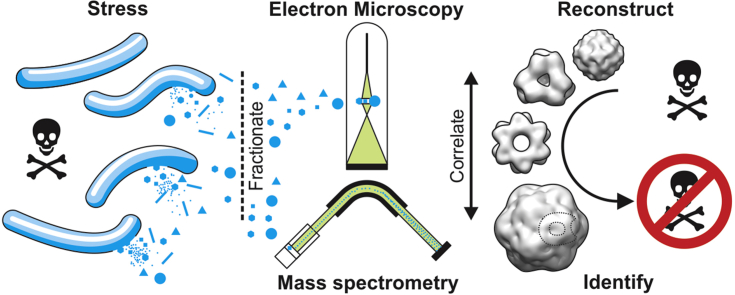

## 1 Introduction

The pathogenic bacterium, *Mycobacterium tuberculosis* (*Mtb*), relies on a range of strategies to evade and manipulate the host immune response (Zhai et al., 2019). Although a large number of *Mtb* persistence mediators have been studied e.g. (Wang et al., 2010; Huo et al., 2015; Sun et al., 2018), structural information is still lacking, particularly for those that form large assemblies. In fact, most protein interactions have only been detected indirectly and there is poor correlation between different detection methods (Mackay et al., 2007). Structures of protein complexes are valuable here, because even at low resolution they provide compelling evidence for their existence. In addition, they can also provide subunit composition, arrangement, and mechanism of interaction, which can yield functional insights (Edwards et al., 2002).

Single-particle transmission electron microscopy (TEM) is a powerful method for the reconstruction of large protein complexes. The technique has been successfully used to solve the structures of endogenous proteins in a range of organisms from homogenous (Han et al., 2009) as well as heterogenous (Maco et al., 2011; Kastritis et al., 2017; Verbeke et al., 2018; Ho et al., 2020) samples. Although this approach offers a faster method for determining the structures of protein complexes without the need for extensive purification (Ho et al., 2020; Kyrilis et al., 2019), it still needs to be tested and adapted for the organism of application (Kastritis et al., 2017; Verbeke et al., 2018; Yi et al., 2019). There is also the problem of identifying protein complexes once they have been reconstructed, which has not been entirely solved for low-resolution data.

Here, we present an adapted shotgun EM methodology for the purification and TEM 3D reconstruction of Mycobacterial protein complexes from the model organism, *M smegmatis* (*Msm*) after exposure to stationary phase stress, which is known to induce a protective effect against subsequent oxidative stress (Smeulders et al., 1999). We combine 3D reconstruction of negatively stained protein complexes and information obtained from mass spectrometry data (shotgun EM) (Verbeke et al., 2018) to efficiently find complexes that could play a role in *Mtb* pathogenesis. This process is dependent on the availability of suitable homologue structures for assigning identity; in the absence of existing models, high-resolution cryo-EM is required to identify the resulting maps (Ho et al., 2020).

We reconstructed and identified four protein complexes (glutamine synthetase I (GSI) (E.C 6.3.1.2), bacterioferritin A (BrfA) (E.C 1.16.3.1), Aspartyl aminopeptidase (apeB) (3.4.11.-) and encapsulin), and demonstrate that encapsulin encloses dye-decolourising type peroxidase (DyP) (E.C 1.11.1.19), an enzymatic anti-oxidant, as its main cargo during stationary phase stress. Furthermore, analysis of our encapsulated DyP shows that it binds on the encapsulin 3-fold axis, validating the relationship between cargo binding and substrate access *in vivo* for *Msm* encapsulin.

## 2 Materials and methods

### 2.1 Culture growth and lysis

*Msm* groELΔC (Noens et al., 2011) was expressed in Middlebrook 7H9 media supplemented with 0.2% glucose, 0.2% glycerol, and 0.05% Tween-80 and grown at 37 °C (120 rpm) to the end of stationary phase (~4–5 days). The cells were pelleted at 4 °C frozen, thawed and resuspended in 25 mL of 50 mM Tris–HCl, 300 mM NaCl, pH 7.2 with protease inhibitor (Roche) and lysed by sonication: 4 × (15 s on, 15 s off for 4 min) on ice. Cell debris was pelleted by centrifugation (20,000 g for 1 h) at 4 °C and filtered (0.45 μm).

### 2.2 Ammonium sulphate precipitation

Ammonium sulphate was slowly added to the filtered supernatant on ice with continual stirring for 30 min before centrifuging at 9,000 g for 15 min. Pellets were clarified by resuspending in 20 mL 50 mM Tris–HCl, 200 mM NaCl, pH 8.0 and centrifuged at 20,000 g for 10 min at 4 °C. The resulting fractions were buffer exchanged into 50 mM Tris–HCl, 200 mM NaCl, pH 8.0 using a centrifugal filter unit with 100 kDa cut-off (Amicon®, Merck, Germany) over several rounds, which also had the effect of excluding small proteins from the sample.

### 2.3 Anion exchange chromatography

The fractions were loaded onto a 20 mL HiPrep Q FF 16/10 column (GE Healthcare Life Sciences, USA) equilibrated with 100–200 mL 20 mM Tris–HCl, 20 mM NaCl, pH 8.0. Weakly bound proteins were excluded by washing with 60 mL of 20 mM Tris–HCl, 0.5 M NaCl, pH 8.0. Proteins were eluted using a gradient of 0.5–1 M NaCl (19.5 CV) at a flow rate of 5 mL/min into 60 fractions and concentrated and buffer exchanged in 50 mM Tris–HCl, 200 mM NaCl, pH 8.0 before use.

### 2.4 Size exclusion chromatography

Samples were loaded onto a gel filtration column (PWXL5000 Tosoh Biosciences, Japan) equilibrated with 50 mM Tris–HCl, 200 mM NaCl, pH 8.0 and eluted at a flow rate of 0.5 mL/min for 1 column volume. Fractions were stored at 4 °C.

### 2.5 Sucrose cushioning

The method applied was adapted from Peyret (2015) with the following modifications: a double sucrose cushion consisting of 25% (top layer) and 70% (bottom layer) sucrose in sodium phosphate buffer (pH 7.4). The sample was centrifuged at 170,462 g for 5 h and the layer just above the 70% cushion was extracted and buffer exchanged as described in 2.3.

### 2.6 Negative stain electron microscopy

Selected samples were pipetted onto glow-discharged (in air, 25 s) continuous carbon-coated copper grids and washed/stained with 5 rounds of 2% uranyl acetate before being air-dried. Images were collected at 2.11- or 3.84 Å/pixel using a Tecnai F20 transmission electron microscope (Phillips/FEI, Netherlands) fitted with a CCD camera (4 k x 4 k) (GATAN US4000 Ultrascan, USA) operated at 200 kV at an electron dose of ~50 e/Å^2^ and a defocus of ~ −1.5 μm.

### 2.7 Classification of particles

Micrographs were imported into Relion 3.1 (Scheres, 2012) without CTF correction. Images were excluded on the basis of astigmatism, poor staining, or noticeable microscope drift. Particles were selected in an unbiased way by reference-free autopicking with Laplacian-of-Gaussian filtering (Zivanov et al., 2018) with a filter diameter range of 10–30 nm. The resulting particles were 2D classified; those classes only containing a small number of particles, poor resolution or multiple separate particles were excluded. Classes with a similar appearance were subjected to further rounds of 2D classification: “*in silico* purification”.

### 2.8 Identifying unique proteins

Two methods were used to identify groups of 2D classes representing identical proteins from different orientations. The first was simple application of “the principle of the brick”: two views of the same 3D object from different orientations will always share at least one dimension. The second is related to the first, but incorporates information about the internal structure of the particle: 2D projections of a 3D object from different orientations will always share a line projection (common line) (Van Heel, 1987). We used SLICEM (Verbeke et al., 2020) to identify this common line with Euclidean scoring and Walktrap clustering and displayed the network with the top N scores (10% of the scores) to identify sets of 2D classes.

### 2.9 Symmetry determination and reconstruction

We assessed the in-plane rotational symmetry of 2D classes and applied particle symmetries that were consistent with all views. Initial maps were generated with Stochastic Gradient Descent and refined using Relion 3.1 3D auto-refine. Incorrect symmetry was identified by poor angular accuracy and subjective evaluation of the map density. Reconstructions were improved by using unsupervised 3D classification to eliminate incorrectly assigned individual images when necessary. UCSF Chimera (Pettersen et al., 2004) was used to display, manipulate and render images.

### 2.10 Molecular weight estimation and model fitting

MW was estimated by adjusting the contour level subjectively to its lower and upper bounds and then applying the relationship: molecular mass (Da) = 825 ∗ V (nm^3^), where V is the volume of the model density at the minimum and maximum contour level. See Erickson (2009) for details on the calculation. The Protein Databank (PDB) (Berman et al., 2000) was searched by subunit molecular mass: (protein assembly molecular mass/stoichiometry) and symmetry. The coordinates were imported into UCSF-Chimera and assessed by docking into the EM maps, map handedness was corrected by inspecting the docking result.

### 2.11 Membrane preparation and electrophoresis

Extracted membranes or anion exchange fractions were analysed by blue or clear native PAGE, to reduce complexity for mass spectrometry analysis. To extract membranes, 2 L of *Msm* culture was grown as described in 2.1 and membranes prepared for blue native PAGE electrophoresis as described previously (Wittig et al., 2006; Zheng et al., 2011). For clear native PAGE a standard continuous Tris-Glycine (pH 8.8) system was used.

### 2.12 Mass spectrometry

Samples were sent for MS either to the Blackburn Group (in-gel native PAGE LC-MS/MS) (University of Cape Town, South Africa) or to the Yale MS & Proteomics Resource (in gel SDS-PAGE LC-MS/MS) (Yale School of Medicine, New Haven, USA). Samples were digested with trypsin and analysed on an LTQ Orbitrap (ThermoScientific, Massachusetts, USA). MS/MS spectra were searched using the Mascot algorithm (Hirosawa et al., 1993). Peaks with a charge state of +2 or +3 were located first using a signal-to-noise ratio of >1.2. Potential peaks were screened against the NCBInr or SWISS-PROT (Bairoch & Apweiler, 2000) databases.

## 3 Results

### 3.1 Establishing a reconstruction workflow

**Fig. 1:**
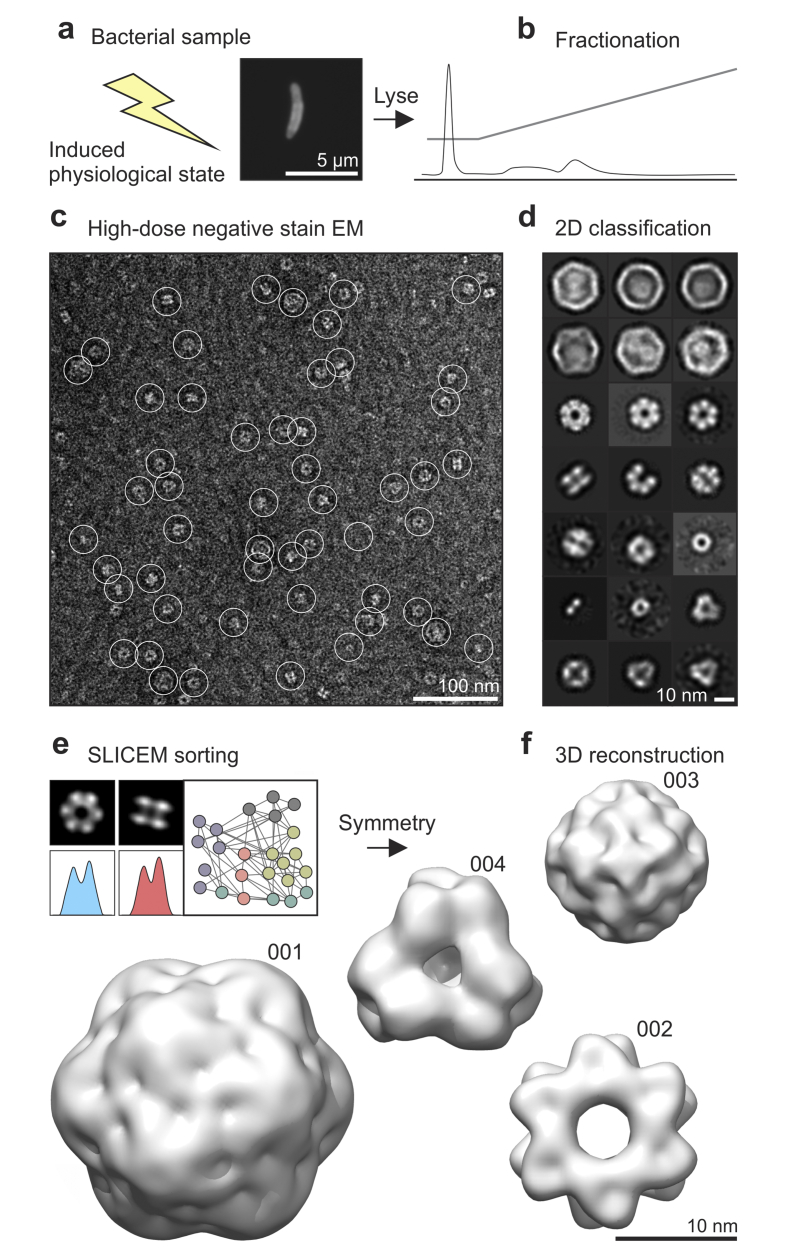
Workflow: partial fractionation, *in silico* purification and identification. a) Cell lysate was collected from late stationary phase *Msm* cells. b) Proteins were fractionated to simplify the identification and reconstruction of protein complexes. c) Uranyl acetate-stained electron micrograph of a filtered anion exchange fraction. Particles were picked in an unbiased way using reference-free autopicking with Laplacian-of-Gaussian filtering (white circles). d) After several rounds of 2D-classification several protein complexes could be seen. e) The 2D classes were sorted into proteins using SLICEM, which identifies the best matching common line and uses this as a score for clustering. In this case, five protein complexes could be sorted into self-consistent views. f) The symmetries of the proteins were estimated from the 2D classes and initial reconstructions generated by stochastic gradient descent and refined in Relion 3.1(Scheres, 2012). Three classes could be reconstructed (001, 002, 004). A fourth protein complex (003) could be reconstructed from a sucrose-cushioning fraction. These four protein complexes could be reconstructed with high certainty from two small datasets (<200 images).

**Fig. 2:**
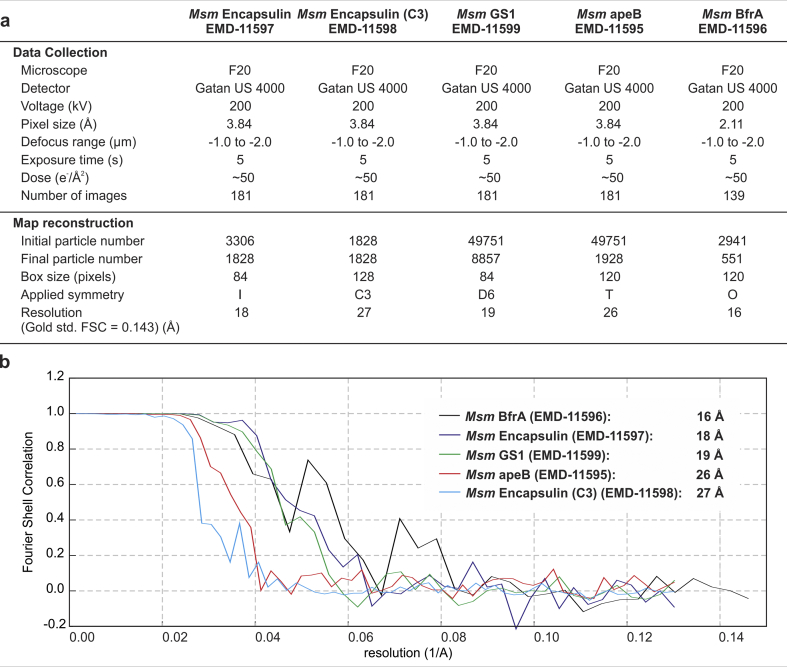
Reconstruction statistics. a) Data collection and processing statistics for the five reconstructions described here. b) Fourier shell correlation plots between two independently reconstructed maps (gold standard), resolutions are quoted at the FSC = 0.143 threshold.

We tested strategies for partial fractionation and reconstruction of *Msm* protein assemblies from cell lysates (Fig. 1a and b). Ammonium sulphate precipitation, ion exchange chromatography, size-exclusion chromatography and sucrose cushion ultracentrifugation were tested in combination with a 100 kDa molecular mass (MW) cut-off and assessed by negative stain EM. In our hands, anion exchange resulted in the best single-step separation in combination with a >100 kDa MW cutoff using a spin concentrator unit (Fig. 1c); sucrose cushioning enriched for a different set of proteins, while the degree of fractionation after ammonium sulphate precipitation was too low to build reliable 3D reconstructions (Supplementary Fig. S1). Fractions were continually assessed by electron microscopy to assess the degree of separation (a total of 67 fractions were screened). Rounds of 2D classification with different mask diameters resulted in *in silico* purified particle views (Fig. 1d), which could be sorted into different protein complexes using SLICEM (Verbeke et al., 2020) (Fig. 1e). Particle symmetries were deduced and imposed after analysing the 2D classification results. Application of this approach led to the 3D reconstruction of four distinct protein complexes (Fig. 1f), reconstruction statistics are provided (Fig. 2).

### 3.2 The complexes were identified using a combined approach

**Fig. 3:**
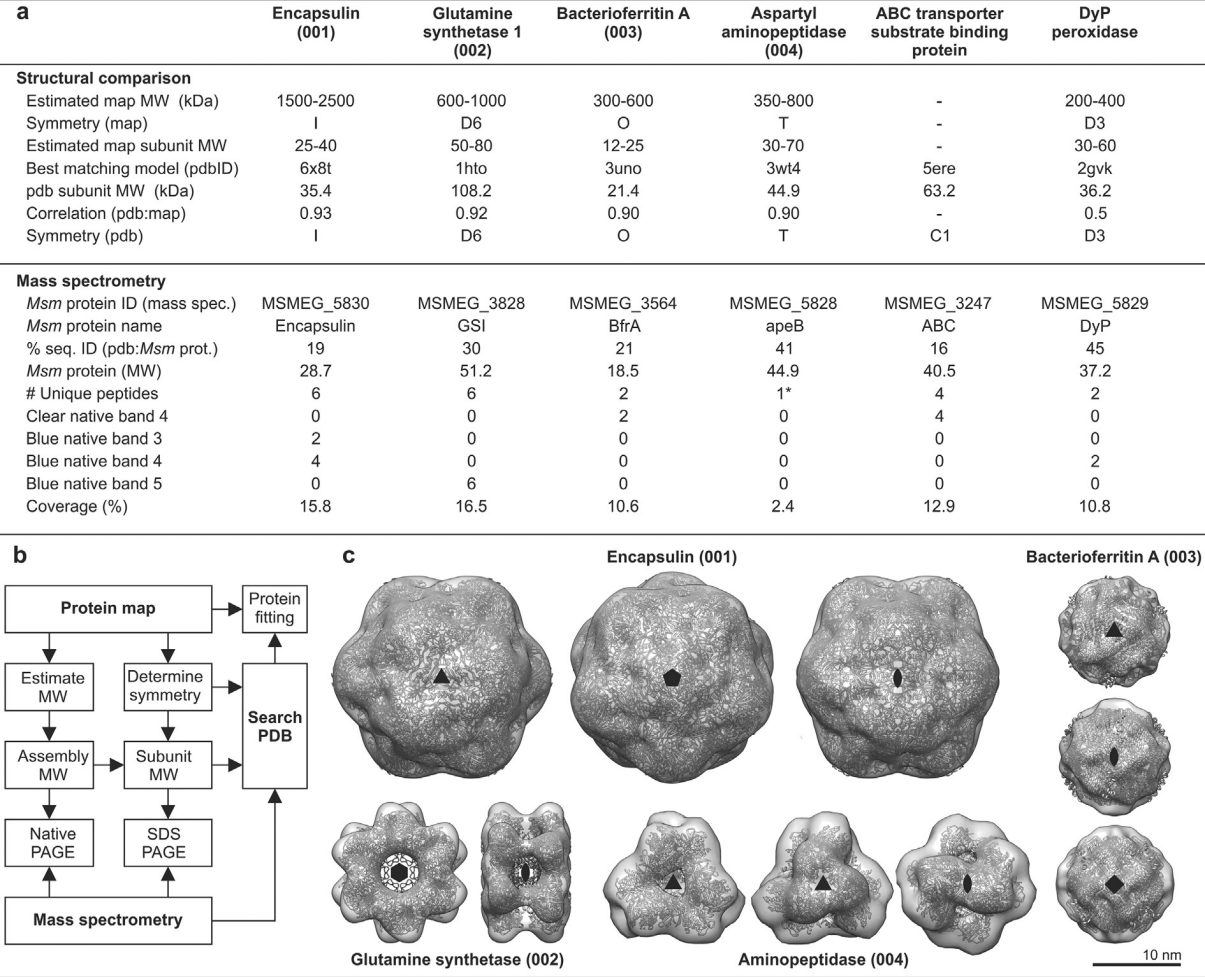
Identification strategy. a) We searched for models in the PDB by symmetry, homology and subunit MW. Bands were excised from clear and blue native gels of solubilised membrane-bound protein and analysed by LC-MS/MS. Single MS/MS peptide hits, or those which were likely to be degraded, or present in controls were manually removed from the analysis. For the full dataset see Supplementary Table S2. Note that increasing band numbers (native bands 3–5) correspond to decreasing MW. b) Overview of the search strategy: the philosophy was to extract as much structural information from our maps as possible and then correlate this to our mass spectrometry hits. c) Atomic models were fitted into our maps: 001: encapsulin from *Synechococcus elongatus* PCC 7942 (pdbID: 6x8t)(LaFrance et al., 2020), and 002: glutamine synthetase I (pdbID: 1hto)(Gill & Eisenberg, 2001), and 003: bacterioferritin A (pdbID: 3uno)(McMath et al., 2011) from *Mycobacterium tuberculosis* and 004: aspartyl aminopeptidase (pdbID: 3wt4)(Nguyen et al., 2014) were identified and docked into the density maps. Crystal structures have good correspondence to the density and symmetry axes. The fit was evaluated by cross-correlation. Symmetry axes are shown for each structure.

We applied a combination of native PAGE, mass spectrometry, molecular mass estimation from the EM model and fitting homologous structures into our maps to identify the protein complexes (Fig. 3a, b, c). Initially, we used the reconstructions themselves to determine the symmetries and estimated subunit MW of the complexes (Fig. 3a), which provided upper and lower bounds for subunit–and complex masses. These were used to help identify PAGE bands, which were analysed by mass spectrometry (Fig. 3a). We searched the PDB (Berman et al., 2000) by symmetry, MW and sequences of the proteins identified to find possible homologues (Fig. 3a and b), which we fitted into our maps (Fig. 3c). The highest matching structures had normalised correlation coefficients of: encapsulin (0.93), glutamine synthase I (GSI) (0.92), bacterioferritin A (BfrA) (0.90) and Aspartyl aminopeptidase (0.90) and fell within the range of our MW estimates. This approach was effective, but had the obvious drawback that it relies on both the availability of a homologue in the PDB and the conservation of its quaternary structure. Furthermore, our approach for estimating MW is only effective if each asymmetric unit only contains one protein.

### 3.3 The encapsulin nanocompartment contained dye-decolourising type peroxidase

**Fig. 4:**
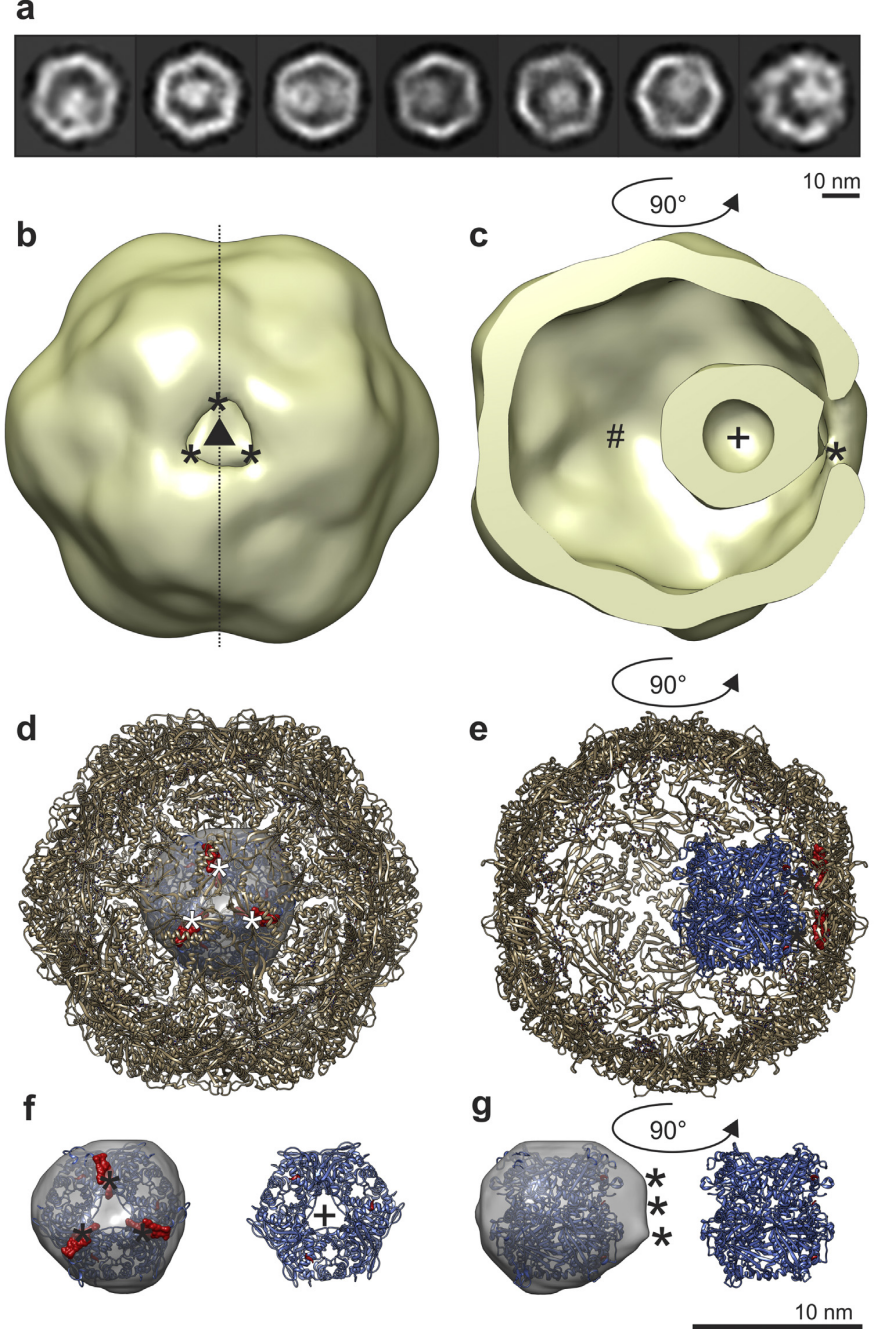
Structure of the primary cargo of *M smegmatis* enapsulin during stationary phase stress. a) Some encapsulin 2D classes appear to show extra density within the nano-compartment, which we suggest belongs to the cargo protein. These particles are ~10 nm in size and, in some cases, show a dark region in their centre. b) After reconstructing encapsulin and applying C3 symmetry, a low-density region is clearly visible in the encapsulin wall (visible as a hole). This is surrounded by three higher density contacts (∗) that connect the particle to the nanocompartment. c) After slicing the map along the midline and rotating it, a clearly defined hollow (+) particle of ~10 nm is size is visible. Contacts between encapsulin and the cargo protein are shown (∗). d) We docked the crystal structure of *Thermotoga maritima* encapsulin (pdbID: 3dkt)(Sutter et al., 2008) into the C3 symmetrized map (correlation coefficient: 0.89), the positions of the contacts (∗) and the DyP localization sequence (red density) exactly superimpose. e) We then docked the crystal structure of a DyP from *Bacteroides thetaiotaomicron* VPI-5482 (pdbID: 2gvk)(Crystal structure of a dye-decolorizing peroxidase (DyP) from Bacteroides thetaiotaomicron VPI-5482 at 1.6 A resolution, 2006) (blue) into the cargo density (correlation coefficient: 0.5), the positions of the C-terminus are indicated in red, while the positions of the *T. maritima* DyP localization sequences are visible as red density. f, g) To visualize the interaction more clearly, we extracted the cargo protein density and indicated the positions of the encapsulin: cargo-protein contacts (∗) and the localization sequence (red density). The size and position of the hollow in the *T. maritima* DyP model corresponds well to the empty density in the core of the cargo protein of our map.

Classified 2D averages of encapsulin particles (Fig. 4a) show density within the nanocompartment, which was icosahedrally averaged in our reconstruction to produce a vague mass. To identify the origin of this density we investigated the literature and found that three *Mtb* proteins have localisation sequences that can direct these proteins into the encapsulin (Rv0798c) nanocompartment when co-expressed recombinantly: dye-decolourising type peroxidase (DyP) (Rv0799c), bacterioferritin B (BfrB) (Rv3841), and 7,8-dihydroneopterin aldolase FolB (Rv3607c) (Contreras et al., 2014). Interestingly, the initial LC-MS/MS data didn't show hits for any of these three proteins (SI Table S1), but both GSI and encapsulin are enriched in Mycobacterial membrane fractions (https://mycobrowser.epfl.ch/) (Kapopoulou et al., 2011). We therefore isolated the membrane fraction of *Msm* and ran the resolubilised material on either blue or clear native PAGE and cut out and analysed all of the visible bands by LC–MS/MS (Fig. 3a, Supplementary Table S2, Supplementary Fig. S2).

We obtained 96 peptide hits after accounting for possible protein degradation (Supplementary Table S2). Both GSI and encapsulin were the highest abundance peptides found in different blue native bands (Fig. 3a). BfrA was also found as a lower abundance peptide in two clear native bands (4 and 5) (Supplementary Table S2, Supplementary Fig. S2), but the only major peptide found exclusively with encapsulin was DyP (Fig. 3a, Supplementary Table S2). To confirm that the cargo protein in our samples was DyP we used EM to identify gel-filtration fractions harbouring encapsulin particles and cargo and separated these by SDS-PAGE. In addition to the encapsulin band, a second lighter band was observed at the expected molecular mass of DyP (~40 kDa). We excised this band and confirmed that DyP was present by mass spectrometry (6.4% coverage) (Supplementary Table S3). Hits that did not match the mass of the band on the gel were excluded from the analysis. None of the other known cargo proteins were observed.

### 3.4 DyP binds on the encapsulin 3-fold axis

We reconstructed the encapsulated cargo by applying C3 symmetry to unmasked particles (Sutter et al., 2008; Putri et al., 2017), which revealed density at the encapsulin 3-fold axis that resembled DyP in size and shape (Fig. 4b and c) (Crystal structure of a dye-decolorizing peroxidase (DyP) from Bacteroides thetaiotaomicron VPI-5482 at 1.6 A resolution, 2006). We estimated the MW of this extra density by segmenting the map in UCSF Chimera and dividing by 6 (D3 symmetry), which gave us the ~expected size of DyP (Fig. 3a). Docking our C3 map, as well as the homologue coordinates, into the icosahedral encapsulin density placed the C-terminal localisation sequence of DyP around the 3-fold encapsulin pore (Fig. 4d and e), which is in agreement with previous studies (Sutter et al., 2008; Putri et al., 2017). We observed co-localization of the three connecting density sites of our map and the localization sequences of the encapsulin model, and reasonable correspondence between these and the C-terminal ends of a docked DyP model (Fig. 4f and g). Only one hexamer can be accommodated in the encapsulin lumen (Fig. 4c), suggesting a molar ratio of 10:1 encapsulin: DyP protein subunits in the fractionated lysate.

## 4 Discussion

### 4.1 The shotgun EM approach

There are fundamental knowledge gaps with regard to the structural biology of the cell. High resolution structures (PDB Statistics, 2020) are only available for about 0.1% of the total sequences in Uniprot (Uniprot Statistics, 2020) and this gap is getting bigger. There is also a strong bias towards monomers and homodimers as these are more amenable to recombinant expression and crystallization (PDB: stoichiometry) (PDB Statistics, 2020). In reality, most proteins function within assemblies of two or more proteins (e.g. Kühner et al. 2009 (Kühner et al., 2009)), but to widely sample this underrepresented portion of the proteome new strategies are needed. Structural analysis of endogenous protein complexes is attractive because it avoids the problems associated with recombinant expression (Kastritis et al., 2017), especially in the case of multi-subunit hetero-complexes. Furthermore, it allows us to reconstruct assemblies whose components are transient or only assemble in a specific physiological state. If we can avoid the time and effort that goes into purification as well, then the shotgun approach seems very appealing.

### 4.2 Fractionation

The reason for fractionating the sample is three-fold: 1) it can enrich for rare proteins that may be crowded out in images, especially very large complexes with low copy numbers; 2) identical objects viewed from different angles may be difficult to group together in impure mixtures, and 3) the identification of reconstructed maps by mass spectrometry is made simpler. Taken to the extreme, samples can be fractionated to homogeneity (Han et al., 2009), this approach is time consuming and limits the number of proteins that can be visualised, but may be pursued to identify completely novel protein complexes. We used a related strategy with encapsulated DyP, by purifying it in different ways and correlating our mass spectrometry results with identification of encapsulin in electron micrographs. Simulated test projections and artificial mixtures of known complexes (Verbeke et al., 2020) have also been used in an effort to simplify the problem. So far, size exclusion chromatography has been the most popular fractionation method (Maco et al., 2011; Kastritis et al., 2017; Verbeke et al., 2018) with selection of high MW fractions because larger proteins are easier to reconstruct by TEM. For the same reason, we imposed a MW cut-off at 100 kDa, but applied anion chromatography to bind proteins and enrich for rare complexes (Ó’Fágáin et al., 2011) (Fig. 1c and d).

### 4.3 *In silico* purification

We selected particles in our micrographs using template-free Laplacian of Gaussian auto-picking and applied rounds of 2D classification in Relion 3.1 in an attempt to eliminate bias resulting from template-based (Verbeke et al., 2018; Verbeke et al., 2020) – or manual picking (Maco et al., 2011; Kastritis et al., 2017; Ho et al., 2020) approaches used previously (Fig. 1d). In our experience, manual picking biases the data towards recognisable and symmetric particles. Template-based picking has the serious disadvantage that the proteins need to be identified previously either by visual inspection of the micrographs or mass spectrometry, where low abundance complexes could potentially be missed in partially fractionated samples (Cottrell, 2011). In addition, there is the risk of “Einstein from noise”: reconstructing the search templates that are actually absent from the images (Henderson, 2013). This is also one reason that we used negative staining with a high electron dose: to obtain the highest signal to noise ratio and reduce the risk of picking spurious particles.

### 4.4 Identifying identical particles in different orientations

2D classification produces a self-consistent set of projections of different proteins from different orientations. These need to be divided into sets representing views of the same object from different directions. This process is straightforward with well-known structures, such as ribosomes (Maco et al., 2011; Kastritis et al., 2017), proteasomes (Maco et al., 2011; Kastritis et al., 2017; Verbeke et al., 2018), and fatty acid synthase (Kastritis et al., 2017), which can be recognised in micrographs and manually picked or picked using a template. Another approach that we attempted, but without success, was 3D classification of all of the 2D classes in Relion 3.1 without imposing symmetry. Although this approach did help us to improve the resolution of reconstructions once 2D classes had been sorted.

An alternative, objective approach is based on the fact that 2D projections of a 3D object share a 1D line-projection, which can be found by comparing the Radon transforms of both projections (Van Heel, 1987). This can form the basis for a classification scheme because the best matching pair can be used to calculate a pairwise score between the two images. We have successfully classified high-pass filtered synthetic projections by calculating the correlation coefficient between pairs of images using Spider v21.11 (Frank et al., 1996). High-pass filtering biases the correlation coefficient towards unique features of the proteins and away from the lowest frequency components, which otherwise dominate the signal. Verbeke et al. (2020) (Verbeke et al., 2020) have used a more refined approach, by calculating the Euclidean distance between 1D projections and clustering 2D classes using these scores. They have made their software available online, which we used here to classify our data. This approach worked for the four complexes described here, but failed for smaller, less distinct 2D classes, especially in the presence of noise. In our case, we obtained the same results using this approach that we did by subjectively selecting particles that looked like they were views of the same object. The automated approach was substantially faster though and could therefore be scaled up.

### 4.5 Symmetry determination and reconstruction

Symmetry was determined by assessing the symmetry of sorted 2D class averages (see Fig. 1, Fig. 3c). Glutamine synthetase shows clear 6-fold symmetry and clear 2-fold symmetry in some 2D classes (when the 3D symmetry axis is perpendicular to the plane of the page) so D6 symmetry was imposed. Likewise, encapsulin shows a clear 3-fold axis and 2-fold axis (Fig. 1, Fig. 3c) but other images appear surprisingly round and featureless (Fig. 1d), which is consistent with both 4-fold and 5-fold symmetries. Octahedral- and icosahedral symmetries were therefore both plausible. However, when we imposed octahedral symmetry this resulted in poor angular assignment in Relion 3.1, as well as inconsistent molecular weight measurements (Fig. 3a). Icosahedral symmetry resulted in a good-quality reconstruction (Fig. 2). BfrA showed noisy 3-fold axis and an obvious 4-fold axis, which implied octahedral symmetry, and apeB had a clear 3-fold and a tilted 3-fold. In the end, symmetries were independently validated by docking structural homologues into our maps (Rosenthal & Rubinstein, 2015), these comparisons also allowed us to determine the correct handedness (Fig. 2, Fig. 3).

### 4.6 Identifying the reconstructed maps

Identification of these initially unknown protein complexes proved to be particularly challenging and we relied on a combination of analysing our maps, LC–MS/MS of native PAGE gel bands and fitting homologues (Fig. 3, Fig. 4). The identification of glutamine synthetase and encapsulin were straightforward because they could be detected in native PAGE (Fig. 3a) and matched their respective docked-structures well (Fig. 3b). BfrA was more difficult because it was detected along with the ABC transporter binding protein by LC-MS/MS in clear native PAGE band 4 (Fig. 3a). However, BfrA homologues fit the map (Fig. 3b) while there is currently no evidence that ABC-transporter substrate binding proteins are octahedral (Hu et al., 2015) (Fig. 3b), with the closest homologue in the pdb being a monomer (pdbID: 5ere) (Cuff et al., 2015). BfrB (MSMEG_6422) is also found in *Msm* and homologues of this protein also have octahedral symmetry, but this is unlikely to be the identity of our structure, because the LC-MS/MS data shows that clear native PAGE band 4 contains BfrA (MSMEG_3564) and not BfrB (Fig. 3a). apeB was detected by LC–MS/MS from SDS-PAGE, albeit with a relatively high expectation score (0.01) (Supplementary Table S4), but the structure was an excellent fit (CC = 0.90) to a crystallized homologue (Fig. 3a and b).

Finding and structurally characterising encapsulated DyP was particularly challenging because our initial mass spectrometry results did not detect it, and its symmetry is mismatched with respect to encapsulin (Icosahedral vs. D3). In retrospect, we suggest that the lack of DyP peptides in this sample to be due to incomplete trypsin digest due to shielding by encapsulin as well as the 1:10 ratio of DyP to encapsulin subunits. We identified it after producing a higher purity encapsulin sample by isolating the membrane fraction and performing LC–MS/MS on blue native PAGE band 4. Both GSI and encapsulin are water-soluble and membrane association may be part of an export process (Tullius et al., 2003; de Souza et al., 2011; V Tullius et al., 2001; Rosenkrands et al., 1998). However, this meant that the reconstruction and mass spectrometry results were from two different samples, so it may be argued that the encapsulins found in soluble lysate might not contain DyP. We do not believe this is the case however, as our C3-imposed encapsulin structure shows a cargo protein the overall size and shape of DyP (Fig. 4f and g). It also forms an operon with encapsulin (Kapopoulou et al., 2011) and has an encapsulin localisation sequence (Supplementary Fig. 3) and DyP is a known encapsulin cargo in other species (Contreras et al., 2014; Sutter et al., 2008; Putri et al., 2017; Nichols et al., 2017).

Other researchers have relied on mass spectrometry data to identify complexes in mixtures (Maco et al., 2011; Kastritis et al., 2017; Verbeke et al., 2018) that have been subjected to TEM, but matching a specific map to a specific protein ID relied on identifying recognizable complexes. Insufficient fractionation hinders this approach (Maco et al., 2011), as does the absence of suitable homologues. An exciting recent development is the demonstration that at better than 4 Å resolution, this problem can be addressed by identifying stretches of amino acids in cryo-EM maps and searching for these sequences in a protein sequence pool derived from genomic sequences (Ho et al., 2020). It will be interesting to see how many proteins can be reconstructed to this resolution from mixed samples.

### 4.7 Encapsulated DyP

Cargo proteins are directed to the encapsulin lumen by symmetrically arranged localisation sequences that bind to similarly positioned binding sites on the inner surface of the nanocompartment (Sutter et al., 2008). On this basis, Sutter et al. (2008) (Sutter et al., 2008) proposed that DyP binds at the 3-fold axis of encapsulin. In *Mtb,* DyP; BfrB and FolB have localisation sequences that direct them into encapsulin when recombinantly expressed (Contreras et al., 2014), but in *Msm* only DyP and BfrB (Khare et al., 2011) have these sequences (Supplementary Fig. S3). On the basis of gel-filtration measurements, Contreras et al. (2014) (Contreras et al., 2014) proposed that *Mtb* DyP forms a mixture of monomers, dimers and tetramers *in vitro*. In contrast, our 2D class averages show a well-resolved particle of ~10 nm in diameter (Fig. 4a), which corresponds more closely to a hexamer and is too small to be BfrB, assuming conservation of its octahedral quaternary structure (Fig. 3a). In 3D, after imposing 3-fold symmetry, this particle was relatively well resolved (Fig. 2, Fig. 4b, c). We interpret this to mean that the particle is centred on the 3-fold axis, which implies that its localisation sequences are symmetrically arranged about the 3-fold axis. This idea is supported by the observation of contacts in our density at three positions corresponding to the binding positions of the localisation sequences (Fig. 4f and g). This is consistent with a hexameric DyP, but it is not clear how this could be achieved in a tetramer. In addition, a channel is clearly visible (Fig. 4c), which is similar to that seen in the D3 symmetric DyP from *Bacteroides thetaiotaomicron* VPI-5482 (pdbID: 2gvk) (Zubieta et al., 2007).

DyP catalyses the oxidation of dyes *in vitro* by catalysing their reaction with H_2_O_2_; *in vivo* its substrates are unknown, but it is thought to act as an antioxidant (Zubieta et al., 2007). *Mtb* DyP retains its activity after encapsulation *in vitro* (Contreras et al., 2014*)*, which suggests that substrates can pass through encapsulin's pores. Interestingly, in our structure the DyP catalytic tunnel is directed towards one of the 3-fold encapsulin pores, which shows significantly reduced density. It is tempting to speculate that this indicates that the pore has changed conformation as a result of DyP binding, activating the pore to allow substrates to be directed into the DyP catalytic site. Higher resolution data will be needed to test this hypothesis. Closely related *B. linens* DyP also binds encapsulin on the 3-fold axis (Putri et al., 2017), which may suggest a common mechanism among the Actinobacteria.

## 5 Conclusions

We reconstructed and identified four protein complexes (encapsulin, GSI, BfrA, and apeB) by ‘shotgun EM’ after exposing *Msm* to stationary phase stress. Several partial fractionation strategies were tested, and the resulting samples were imaged by negative stain EM. We applied an unbiased picking, 2D classification, and sorting approach. Identification of these initially unknown protein complexes proved to be particularly challenging and relied on a combination of LC–MS/MS of native PAGE gel bands and fitting of homologues crystal structures. Under stationary phase stress, *Msm* encapsulin appears to primarily enclose DyP, a protein antioxidant. Production of these complexes may have functional significance in *Msm*, as one of the mechanisms by which it develops resistance to oxidative stress after growth in stationary phase. These results demonstrate the utility of applying a ‘shotgun EM’ methodology to identify previously uncharacterised protein complexes that may play vital roles in the ability of *Mtb* to survive and reproduce in the hostile environment of the host.

## 6 Data availability

All maps have been deposited in the Electron Microscopy Data Bank (https://www.ebi.ac.uk/pdbe/emdb/) (Tagari et al., 2002): *Msm* encapsulin (MSMEG_5830): EMD-11597; *Msm encapsulin* with DyP type peroxidase (MSMEG_5829) bound on 3-fold axis: EMD-11598; *Msm* glutamine synthetase (MSMEG_3828): EMD-11599; *Msm* aspartyl aminopeptidase (MSMEG_5828): EMD-11595 and *Msm* bacterioferritin A (MSMEG_3564): EMD-11596. Protein sequences are available from Mycobrowser (https://mycobrowser.epfl.ch/) (Kapopoulou et al., 2011).

## CRediT authorship contribution statement

**Angela M. Kirykowicz:** Investigation, Writing - original draft. **Jeremy D. Woodward:** Conceptualization, Resources, Funding acquisition, Supervision, Visualization, Writing - review & editing.

## Declaration of competing interest

The authors declare that they have no known competing financial interests or personal relationships that could have appeared to influence the work reported in this paper.
