# Supplementary material for: Shotgun EM of mycobacterial protein complexes during stationary phase stress
Source: Curr Res Struct Biol. 2020 Sep 22;2:204–12. doi: 10.1016/j.crstbi.2020.09.002 (PMC8244302; doi:10.1016/j.crstbi.2020.09.002)
Supplement: Multimedia component 1 [file mmc1.docx]

**Supplementary Information: Shotgun EM of Mycobacterial protein complexes during stationary phase stress.**

**Authors:** Angela M Kirykowicz^1,2+^ & Jeremy D Woodward^2^*

**Affiliations:**

^1^Current affiliation: Department of Biochemistry, University of Cambridge, Sanger Building, Tennis Court Road, Cambridge, CB2 1GA

^2^Division of Medical Biochemistry and Structural Biology, Department of Integrative Biomedical Sciences, University of Cape Town, Anzio Road, Observatory 7925, Cape Town, South Africa and Structural Biology Research Unit, University of Cape Town.

^*^Corresponding author: [jeremydavidwoodward@gmail.com](mailto:jeremydavidwoodward@gmail.com)

**Keywords: three dimensional electron microscopy (3DEM), protein structure, mycobacteria, oxidative stress, structural proteomics, shotgun approach**


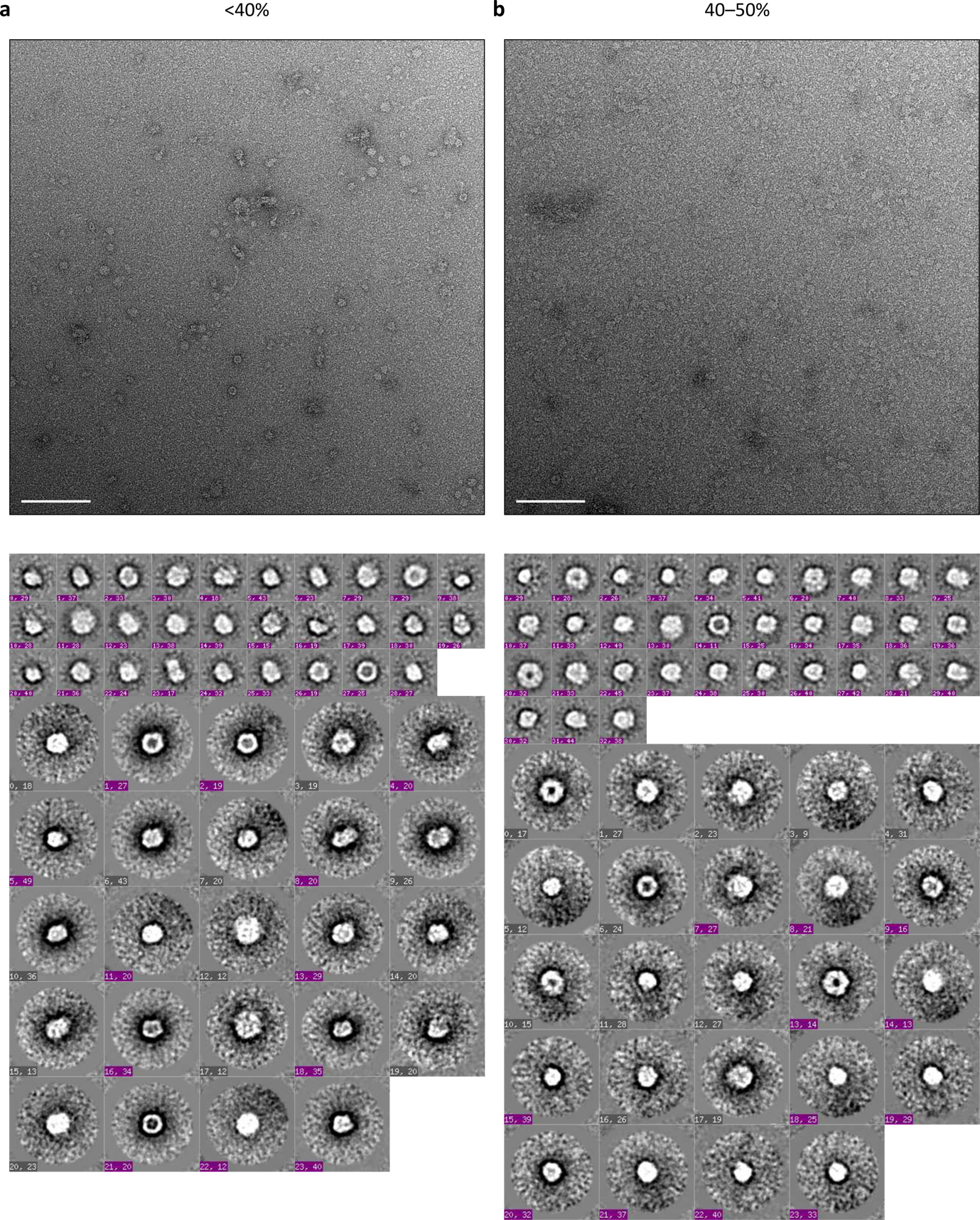


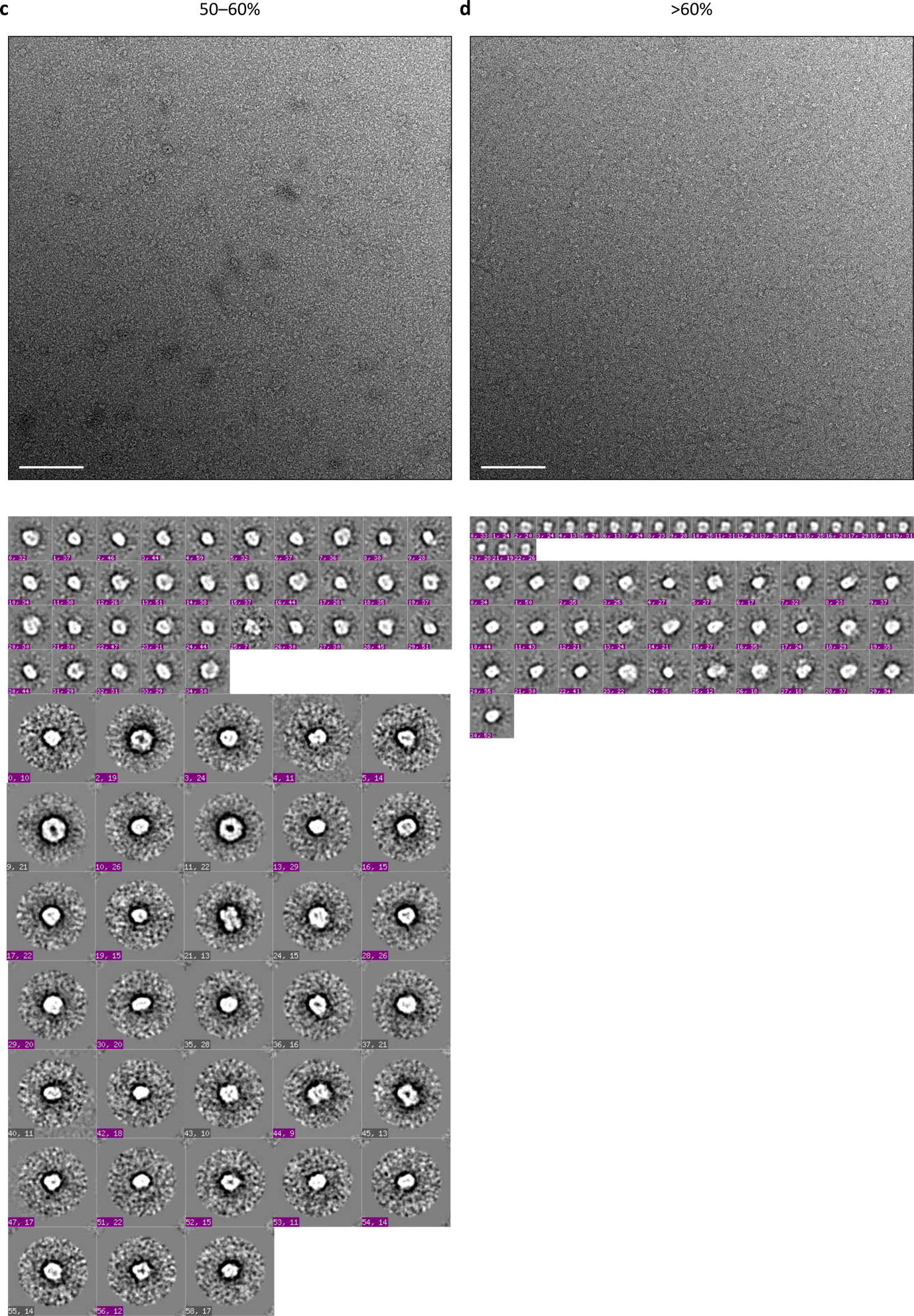


**Figure S1 (previous page). Diversity of protein complexes in *M smegmatis*.** Cell lysate was fractionated by a) <40%, b) 40−50%, c) 50−60%, and d) >60% ammonium sulphate cuts (top row). Particles were picked and assigned to class averages using multivariate statistics through the processing pipeline Appion (bottom row) (Lander et al., 2009). Images were taken at x50,000 magnification at a defocus of 2.00 μm using an F20 Tecnai TEM. Scale bars (white) show 100 nm.


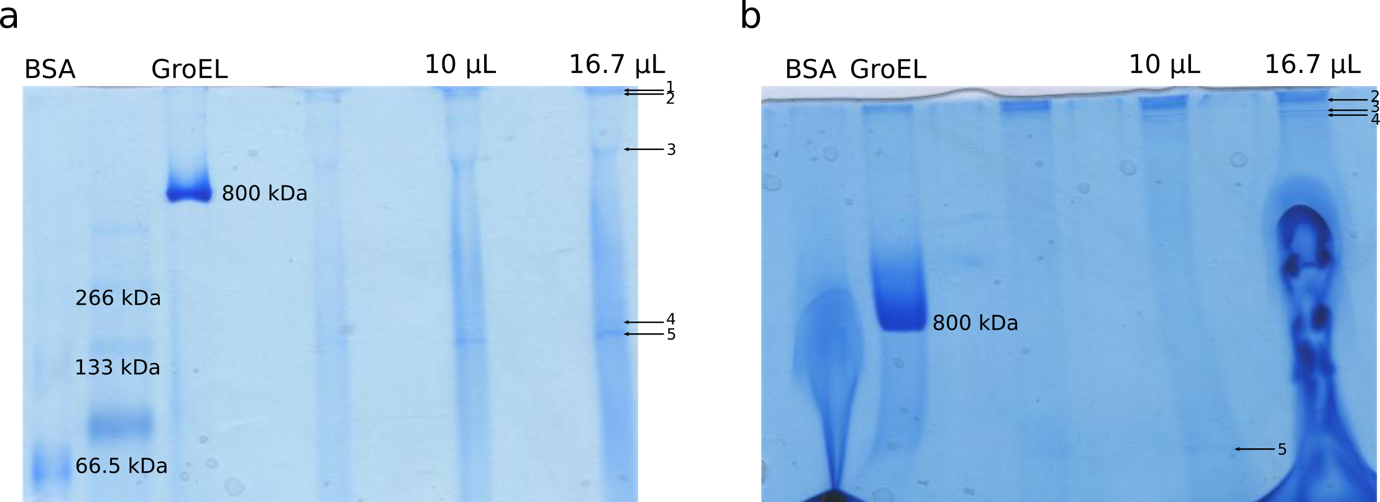


**Figure S2. Analysis of *Msm* membrane fraction**. 7% resolving clear (a) and blue (b) native PAGE was run for 10 µL or 16.7 µL re-solubilised membrane. Commercial BSA and GroEL (Sigma-Aldrich, 1 mg/mL) show approximate MW estimates. BSA predominantly exists in monomeric form, but small amounts of higher-order oligomers are also present (Ramjeesingh *et al*, 1999). BSA sample did not run successfully on blue native PAGE. Bands 1−4 and 2−5 for respective clear and blue native PAGE gels were cut out and sent for LC−MS/MS analysis.


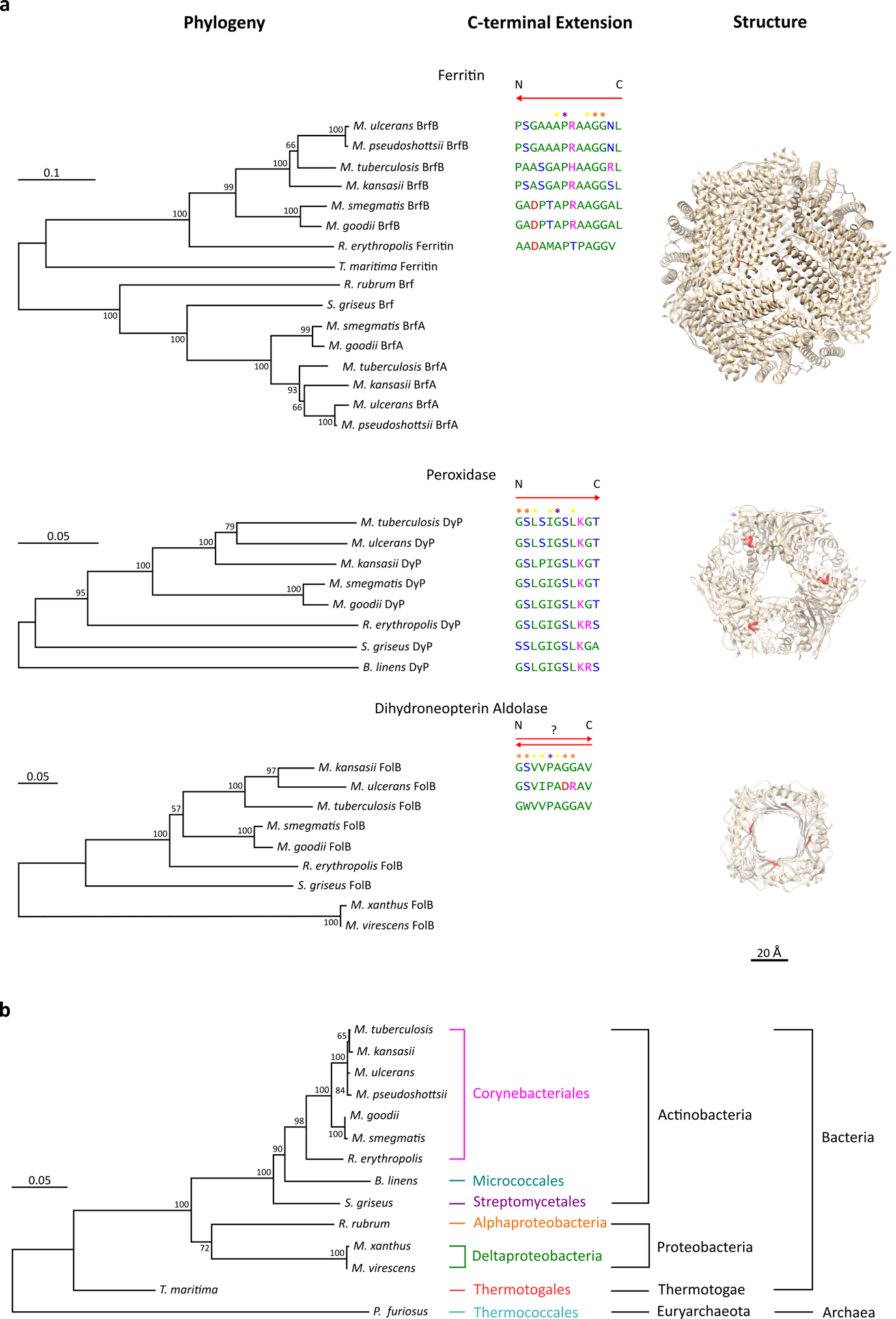


**Figure S3 (previous page). Cargo proteins of encapsulin. A)** The phylogeny, C-terminal extension, and structure are given for the three known cargo proteins of *Mtb*. Binding of the cargo protein to the inside of encapsulin is determined by the C-terminal extension, which is dominated by non-polar amino acids (green) with interspersed with mostly conserved polar (blue), positively charged (pink), or negatively charged (red) amino acids. The direction of binding is determined by two N- or C-terminal residues (orange star) while a central residue (purple star) separating two hydrophobic residues (yellow star) aids in positioning (Sutter et al., 2008). The direction of binding for the 7,8-dihydroneopterin aldolase cargo is ambiguous. Binding of the C-terminal extension (red) is hypothesised to occur along either the 3-fold or 4-fold axis of the cargo protein. Ferritin cargo protein may also bind along its 2-fold axis (not shown). Note that the C-terminal extension is only visible for *Mtb* ferritin (pdb 3uno (McMath et al., 2012)) and was not built into the crystal structure of peroxidase (pdb code 2gvk (JCSG, 2006)) and was cleaved from 7,8-dihydroneopterin aldolase (pdb code 1nbu (Goulding et al., 2004)). Also note that the peroxidase shown is from the closest structural homolog, *Bacteroides thetaiotaomicron*. **B)** Phylogenetic relationship between organisms that harbour known and putative encapsulin and cargo proteins, based on 16S rRNA gene sequence. While the peroxidase cargo is found in the Actinobacteria phylum, the ferritin cargo is restricted to the Corneybacteriales order, and the 7,8-dihydroneopterin aldolase cargo is specific to slow-growing Mycobacteria. For the phylogenetic trees, scale bars show amino acid or nucleotide substitutions.

**SI Methods**

**Phylogenetic Analysis**

Alignments of protein sequences were produced in UCSF-Chimera (Petterson et al., 2004) and exported to MEGA6 (Tamura et al., 2013) for phylogenetic analysis. Alignment of DNA sequences was completed in MEGA6 using MUSCLE (Edgar et al., 2004) with default parameters. For protein sequences, a neighbour joining-tree was produced using p-distance to model amino acid substitution; the rate of substitution was assumed to be uniform and the pattern among lineages homogenous; gaps or missing data were deleted in the analysis. For DNA sequences, a minimal evolution tree was constructed using p-distance to model nucleotide substitutions; only transitions were included while the rate of substitution was assumed to be uniform and homogenous across lineages; gaps or missing data were deleted from the analysis. Trees were bootstrapped using 1000 replicates.

**Supplementary Information References**

Edgar, R.C. 2004. MUSCLE: multiple sequence alignment with high accuracy and high throughput. Nucleic Acids Res. 32(5), 1792-7.

[dataset] Goulding, C.W., Apostol, M.I., Sawaya, M.R., Phillips, M., Parseghian, A., Eisenberg, D., TB Structural Genomics Consortium (TBSGC). 2004. 7,8-Dihydroneopterin Aldolase Complexed with Product From Mycobacterium Tuberculosis. Protein Data Bank. 1NBU.

[dataset] Joint Center for Structural Genomics (JCSG). (2006) Crystal structure of a dye-decolorizing peroxidase (DyP) from Bacteroides thetaiotaomicron VPI-5482 at 1.6 A resolution. Protein Data Bank. 2GVK.

Lander, G.C., Stagg, S.M., Voss, N.R., Cheng, A., Fellmann, D., Pulokas, J., Yoshioka, C., Irving, C., et al. 2009. Appion: an integrated, database-driven pipeline to facilitate EM image processing. Journal of Structural Biology. 166(1): 95-102.

[dataset] McMath, L.M., Contreras, H., Goulding, C.W., TB Structural Genomics Consortium (TBSGC). 2012. Mycobacterium tuberculosis ferritin homolog, BfrB. 3UNO.

Ramjeesingh, M., Huan, L.J., Garami, E., Bear, C.E. 1999. Novel method for evaluation of the oligomeric structure of membrane proteins. Biochemical Journal. 342: 119-23.

Sutter, M., Boehringer, D., Gutmann, S., Günther, S., Prangishvili, D., Loessner, M.J., Stetter, K.O., Weber-Ban, E., et al. 2008. Structural basis of enzyme encapsulation into a bacterial nanocompartment. Nature Structural & Molecular Biology. 15(9): 939-47.

Tamura, K., Stecher, G., Peterson, D., Filipski, A., Kumar, S. 2013. MEGA6: Molecular Evolutionary Genetics Analysis Version 6.0. Mol. Biol. Evol. 30(12), 2725-9.

**Supplementary Table S1. LC-MS/MS native PAGE anion exchange fractions**

| **Protein IDs** | **Uniprot ID** | **Unique peptides 700kDa** | **Unique peptides >1MDa** | **Unique sequence coverage [%]** | **Mol. weight [kDa]** | **Q-value** | **Score** | **MS/MS count** |
| --- | --- | --- | --- | --- | --- | --- | --- | --- |
| A0QS98 | Elongation factor Tu | 1 | 0 | 6.8 | 43.735 | 0 | 126.56 | 37 |
| A0R079 | Glutamine synthetase | 12 | 0 | 27.8 | 53.591 | 0 | 99.681 | 30 |
| A0R0X1 | Major membrane protein I | 0 | 4 | 20.2 | 33.727 | 0 | 30.681 | 7 |
| A0R4H0 | 29 kDa antigen Cfp29 | 0 | 6 | 29.4 | 28.73 | 0 | 176.48 | 45 |

**Supplementary Table S2. LC-MS/MS clear and blue native PAGE**

| **Majority protein IDs** | **Peptide counts (unique)** | **Uniprot ID** | **Number of proteins** | **Unique peptides** | **Unique peptides Clear Band 1** | **Unique peptides Clear Band 2** | **Unique peptides Clear Band 3** | **Unique peptides Clear Band 4** | **Unique peptides Clear Band 5** | **Unique peptides Blue Band 2** | **Unique peptides Blue Band 3** | **Unique peptides Blue Band 4** | **Unique peptides Blue Band 5** | **Unique sequence coverage [%]** | **Mol. weight [kDa]** | **Q-value** | **Score** | **MS/MS count** |
| --- | --- | --- | --- | --- | --- | --- | --- | --- | --- | --- | --- | --- | --- | --- | --- | --- | --- | --- |
| A0QNE2 | 2 | Putative conserved membrane protein | 1 | 2 | 0 | 0 | 0 | 1 | 2 | 0 | 0 | 0 | 0 | 10.4 | 27.228 | 0 | 28.679 | 3 |
| A0QNN8;A0QZQ3 | 2;1 | NAD(P) transhydrogenase alpha subunit | 2 | 2 | 0 | 0 | 0 | 2 | 2 | 0 | 0 | 0 | 0 | 4.7 | 52.706 | 0 | 13.488 | 4 |
| A0QP93 | 3 | Uncharacterized protein | 1 | 3 | 0 | 0 | 0 | 3 | 3 | 0 | 0 | 0 | 0 | 12.8 | 43.259 | 0 | 31.047 | 8 |
| A0QPE7 | 8 | 3-oxoacyl-acyl-carrier protein reductase FabG4 | 1 | 8 | 0 | 0 | 0 | 2 | 8 | 0 | 0 | 0 | 0 | 24.9 | 46.498 | 0 | 86.507 | 10 |
| A0QPE8 | 2 | 3-ketoacyl-CoA thiolase | 1 | 2 | 0 | 0 | 0 | 2 | 1 | 0 | 0 | 0 | 0 | 9.3 | 45.233 | 0 | 16.873 | 3 |
| A0QPJ3 | 1 | Succinate dehydrogenase flavoprotein subunit | 1 | 1 | 0 | 0 | 0 | 1 | 1 | 0 | 0 | 0 | 0 | 1.7 | 70.055 | 0 | 8.8439 | 2 |
| A0QPX3 | 4 | Aliphatic sulfonates family ABC transporter | 1 | 4 | 0 | 0 | 0 | 4 | 4 | 0 | 0 | 0 | 0 | 17.4 | 34.503 | 0 | 47.048 | 8 |
| A0QQ81 | 1 | Bacterial extracellular solute-binding protein | 1 | 1 | 0 | 0 | 0 | 1 | 1 | 0 | 0 | 0 | 0 | 3.5 | 46.499 | 0.0033003 | 5.9233 | 2 |
| A0QQH1 | 2 | Uncharacterized protein | 1 | 2 | 0 | 0 | 0 | 1 | 2 | 0 | 0 | 0 | 0 | 13.2 | 28.976 | 0 | 15.041 | 5 |
| A0QQU5 | 7 | 60 kDa chaperonin 1 | 1 | 7 | 0 | 0 | 0 | 3 | 6 | 2 | 0 | 0 | 0 | 16.3 | 56.487 | 0 | 59.801 | 14 |
| A0QQW8 | 2 | Dihydrolipoyl dehydrogenase | 1 | 2 | 0 | 0 | 0 | 2 | 2 | 0 | 0 | 0 | 0 | 5 | 49.469 | 0 | 13.941 | 3 |
| A0QR46 | 3 | Immunogenic protein | 1 | 3 | 0 | 0 | 0 | 2 | 2 | 0 | 0 | 0 | 0 | 12.3 | 36.237 | 0 | 43.958 | 4 |
| A0QR51 | 1 | Uncharacterized protein | 1 | 1 | 0 | 0 | 1 | 0 | 0 | 1 | 0 | 0 | 0 | 18.7 | 13.038 | 0 | 7.7741 | 2 |
| A0QR99 | 3 | FEIII-dicitrate-binding periplasmic lipoprotein FecB | 1 | 3 | 0 | 0 | 0 | 1 | 3 | 0 | 0 | 0 | 0 | 14 | 36.903 | 0 | 35.203 | 5 |
| A0QRS0 | 2 | ABC-type transport system periplasmic substrate-binding protein | 1 | 2 | 0 | 0 | 0 | 2 | 2 | 0 | 0 | 0 | 0 | 6.7 | 44.535 | 0 | 16.35 | 5 |
| A0QS72 | 3 | Ribose ABC transporter, periplasmic binding protein | 1 | 3 | 0 | 0 | 0 | 3 | 2 | 0 | 0 | 0 | 0 | 16.1 | 35.638 | 0 | 29.686 | 8 |
| A0QS98 | 2 | Elongation factor Tu | 1 | 2 | 0 | 0 | 0 | 1 | 1 | 2 | 0 | 0 | 0 | 7.1 | 43.735 | 0 | 20.566 | 4 |
| A0QSG8 | 2 | 50S ribosomal protein L15 | 1 | 2 | 0 | 0 | 0 | 0 | 0 | 2 | 1 | 0 | 0 | 19.7 | 15.571 | 0 | 32.269 | 5 |
| A0QSL1 | 4 | SPFH domain / Band 7 family protein | 1 | 4 | 0 | 0 | 1 | 0 | 0 | 4 | 0 | 0 | 0 | 11.4 | 54.604 | 0 | 26.653 | 6 |
| A0QSL5 | 2 | 30S ribosomal protein S13 | 1 | 2 | 0 | 0 | 0 | 0 | 0 | 2 | 0 | 0 | 0 | 18.5 | 14.217 | 0 | 13.92 | 2 |
| A0QSY1 | 2 | ABC transporter, ATP-binding protein | 1 | 2 | 0 | 0 | 0 | 0 | 0 | 2 | 0 | 0 | 0 | 3 | 95.528 | 0 | 13.364 | 2 |
| A0QSZ3 | 12 | Isocitrate dehydrogenase (NADP) Icd2 | 1 | 12 | 0 | 0 | 0 | 12 | 6 | 1 | 0 | 0 | 0 | 19 | 82.603 | 0 | 130.26 | 31 |
| A0QT21 | 1 | Cytosine/purine/uracil/thiamine/allantoin permease family protein | 1 | 1 | 0 | 0 | 0 | 1 | 1 | 0 | 0 | 0 | 0 | 2.5 | 51.015 | 0 | 6.3694 | 2 |
| A0QT42 | 7 | ABC transporter | 1 | 7 | 0 | 0 | 0 | 5 | 6 | 0 | 0 | 0 | 0 | 27.7 | 38.767 | 0 | 51.32 | 9 |
| A0QT50 | 8 | ABC transporter periplasmic-binding protein YtfQ | 1 | 8 | 0 | 0 | 0 | 6 | 7 | 0 | 0 | 0 | 0 | 26.2 | 34.234 | 0 | 132.87 | 22 |
| A0QTE1 | 6 | Acetyl-/propionyl-coenzyme A carboxylase alpha chain | 1 | 6 | 0 | 0 | 0 | 3 | 6 | 0 | 0 | 0 | 0 | 12.5 | 63.137 | 0 | 44.999 | 8 |
| A0QU82 | 1 | Secreted protein | 1 | 1 | 0 | 0 | 0 | 1 | 1 | 0 | 0 | 0 | 0 | 5.2 | 21.997 | 0 | 6.7448 | 2 |
| A0QVB8 | 3 | 30S ribosomal protein S2 | 1 | 3 | 0 | 0 | 0 | 0 | 2 | 1 | 0 | 0 | 0 | 14.1 | 30.107 | 0 | 24.273 | 3 |
| A0QVL2 | 2 | Probable malate:quinone oxidoreductase | 1 | 2 | 0 | 0 | 0 | 0 | 1 | 1 | 0 | 0 | 0 | 5.7 | 54.913 | 0 | 12.112 | 2 |
| A0QVX3 | 6 | Glutamate binding periplasmic protein | 1 | 6 | 0 | 0 | 0 | 6 | 2 | 0 | 0 | 0 | 0 | 23.8 | 29.036 | 0 | 109.81 | 10 |
| A0QW16 | 2 | Conserved alanine valine and glycine rich protein | 1 | 2 | 0 | 0 | 0 | 0 | 0 | 2 | 0 | 0 | 0 | 5 | 42.534 | 0 | 11.345 | 2 |
| A0QW29 | 1 | Hydrolase, alpha/beta fold family protein | 1 | 1 | 0 | 0 | 0 | 1 | 1 | 0 | 0 | 0 | 0 | 1.7 | 54.607 | 0.0033333 | 5.9868 | 2 |
| A0QWJ2 | 3 | Protein translocase subunit SecD | 1 | 3 | 0 | 0 | 3 | 0 | 0 | 0 | 0 | 0 | 0 | 7.3 | 63.646 | 0 | 18.174 | 3 |
| A0QWJ4 | 1 | Bacterial extracellular solute-binding protein, family protein 5 | 1 | 1 | 0 | 0 | 0 | 1 | 1 | 0 | 0 | 0 | 0 | 2.2 | 58.2 | 0 | 6.4874 | 2 |
| A0QWL3 | 2 | Extracellular ligand-binding receptor | 1 | 2 | 0 | 0 | 0 | 2 | 0 | 0 | 0 | 0 | 0 | 6.2 | 45.299 | 0 | 63.776 | 2 |
| A0QWT6 | 2 | Lipoprotein | 1 | 2 | 0 | 0 | 1 | 1 | 1 | 0 | 0 | 0 | 0 | 7.9 | 30.577 | 0 | 11.986 | 2 |
| A0QX98 | 1 | Prolipoprotein diacylglyceryl transferase | 1 | 1 | 0 | 0 | 1 | 0 | 0 | 0 | 0 | 0 | 0 | 2.9 | 65.041 | 0 | 8.3142 | 2 |
| A0QXA5 | 1 | Uncharacterized protein | 1 | 1 | 0 | 0 | 0 | 1 | 1 | 0 | 0 | 0 | 0 | 11 | 27.25 | 0 | 11.146 | 4 |
| A0QXA9 | 1 | Integral membrane protein | 1 | 1 | 0 | 0 | 0 | 1 | 1 | 1 | 0 | 0 | 0 | 8.9 | 19.563 | 0 | 6.2415 | 3 |
| A0QXB0 | 4 | ABC-type amino acid transport system, secreted component | 1 | 4 | 0 | 0 | 0 | 4 | 4 | 0 | 0 | 0 | 0 | 20 | 30.706 | 0 | 174.48 | 19 |
| A0QXC0 | 4 | Branched-chain amino acid ABC transporter substrate-binding protein | 1 | 4 | 0 | 0 | 0 | 4 | 0 | 0 | 0 | 0 | 0 | 12.9 | 40.502 | 0 | 35.926 | 4 |
| A0QXJ8 | 1 | Uncharacterized protein | 1 | 1 | 0 | 0 | 0 | 1 | 1 | 0 | 0 | 0 | 0 | 11.3 | 14.853 | 0 | 80.726 | 3 |
| A0QY79 | 2 | Bacterioferritin | 1 | 2 | 0 | 0 | 0 | 2 | 1 | 0 | 0 | 0 | 0 | 10.6 | 18.482 | 0 | 22.679 | 3 |
| A0QYB5 | 5;1 | D-threitol-binding protein | 2 | 5 | 0 | 0 | 0 | 2 | 4 | 0 | 0 | 0 | 0 | 20.6 | 36.35 | 0 | 34.418 | 5 |
| A0QYF6 | 6 | von Willebrand factor, type A | 1 | 6 | 0 | 0 | 0 | 4 | 4 | 0 | 0 | 0 | 0 | 11.9 | 80.66 | 0 | 47.838 | 8 |
| A0QYH0 | 2 | ABC transporter domain protein | 1 | 2 | 0 | 0 | 0 | 1 | 2 | 0 | 0 | 0 | 0 | 4.4 | 71.633 | 0 | 13.187 | 4 |
| A0QZ37 | 1 | Uncharacterized protein | 1 | 1 | 0 | 0 | 0 | 1 | 0 | 0 | 0 | 0 | 0 | 6.6 | 30.66 | 0 | 7.3859 | 3 |
| A0QZ56 | 2 | LppK | 1 | 2 | 0 | 0 | 0 | 0 | 2 | 0 | 0 | 0 | 0 | 15 | 19.244 | 0 | 15.926 | 2 |
| A0QZY7 | 3 | Lipoprotein lppL | 1 | 3 | 0 | 0 | 0 | 1 | 2 | 0 | 0 | 0 | 0 | 11.3 | 35.069 | 0 | 20.097 | 2 |
| A0QZZ9 | 4 | Secreted protein | 1 | 4 | 0 | 0 | 0 | 4 | 3 | 1 | 0 | 0 | 0 | 17.1 | 34.566 | 0 | 64.433 | 15 |
| A0R006 | 3 | Cell wall synthesis protein Wag31 | 1 | 3 | 0 | 0 | 1 | 0 | 0 | 0 | 1 | 1 | 0 | 16.2 | 29.544 | 0 | 17.749 | 3 |
| A0R050 | 1 | Ubiquinol-cytochrome c reductase cytochrome c subunit | 1 | 1 | 0 | 0 | 0 | 0 | 1 | 1 | 0 | 0 | 0 | 6.7 | 27.846 | 0 | 16.773 | 2 |
| A0R069 | 3 | Probable cytosol aminopeptidase | 1 | 3 | 0 | 0 | 3 | 0 | 0 | 2 | 0 | 0 | 0 | 6.9 | 53.724 | 0 | 24.434 | 7 |
| A0R079 | 6 | Glutamine synthetase 1 | 1 | 6 | 0 | 0 | 0 | 0 | 0 | 0 | 0 | 0 | 6 | 16.5 | 53.591 | 0 | 51.693 | 9 |
| A0R085 | 3 | Protease | 1 | 3 | 0 | 0 | 0 | 2 | 2 | 0 | 0 | 0 | 0 | 6.9 | 54.106 | 0 | 20.162 | 4 |
| A0R0W7 | 6 | Sulfate-binding lipoprotein SubI | 1 | 6 | 0 | 0 | 0 | 6 | 3 | 0 | 0 | 0 | 0 | 24.4 | 36.683 | 0 | 58.321 | 17 |
| A0R0X2 | 6 | Cysteine desulfurase | 1 | 6 | 2 | 0 | 0 | 0 | 0 | 0 | 6 | 1 | 0 | 14.1 | 59.621 | 0 | 49.418 | 14 |
| A0R183 | 4 | Sugar ABC transporter substrate-binding protein | 1 | 4 | 0 | 0 | 0 | 4 | 4 | 0 | 0 | 0 | 0 | 16.1 | 34.122 | 0 | 51.737 | 12 |
| A0R1A4 | 4 | Uncharacterized protein | 1 | 4 | 0 | 0 | 0 | 4 | 4 | 0 | 0 | 0 | 0 | 18.8 | 27.96 | 0 | 32.318 | 13 |
| A0R1D2 | 2 | Dihydrolipoamide acetyltransferase component of pyruvate dehydrogenase complex | 1 | 2 | 0 | 0 | 0 | 0 | 0 | 2 | 0 | 0 | 0 | 6.4 | 42.841 | 0 | 12.796 | 2 |
| A0R1H7 | 4 | Fatty acid synthase | 1 | 4 | 0 | 0 | 3 | 0 | 0 | 1 | 0 | 0 | 0 | 1.6 | 329.53 | 0 | 26.819 | 4 |
| A0R1Z9 | 3 | ATP synthase epsilon chain | 1 | 3 | 0 | 0 | 0 | 0 | 3 | 0 | 0 | 0 | 0 | 33.9 | 13.265 | 0 | 19.551 | 4 |
| A0R201 | 6 | ATP synthase gamma chain | 1 | 6 | 0 | 0 | 0 | 0 | 6 | 0 | 0 | 0 | 0 | 24.4 | 33.397 | 0 | 174.09 | 18 |
| A0R261 | 3 | Bacterial extracellular solute-binding protein, family protein 5 | 1 | 3 | 0 | 0 | 0 | 3 | 3 | 0 | 0 | 0 | 0 | 5.8 | 61.075 | 0 | 28.092 | 7 |
| A0R280 | 2 | Lipoprotein | 1 | 2 | 0 | 0 | 0 | 2 | 1 | 0 | 0 | 0 | 0 | 10 | 21.659 | 0 | 17 | 3 |
| A0R2B0 | 1 | Putative membrane protein | 1 | 1 | 0 | 0 | 0 | 1 | 1 | 0 | 0 | 0 | 0 | 5.5 | 26.261 | 0 | 7.1913 | 2 |
| A0R2C0 | 2 | ABC transporter, ATP-binding protein SugC | 1 | 2 | 0 | 0 | 0 | 1 | 1 | 0 | 0 | 0 | 0 | 4.9 | 43.656 | 0 | 12.997 | 2 |
| A0R2C2 | 1 | ABC sugar transporter, permease component | 1 | 1 | 0 | 0 | 0 | 1 | 1 | 0 | 0 | 0 | 0 | 5.2 | 32.719 | 0 | 12.563 | 2 |
| A0R2C3 | 4 | Bacterial extracellular solute-binding protein | 1 | 4 | 0 | 0 | 0 | 4 | 2 | 0 | 0 | 0 | 0 | 12 | 50.14 | 0 | 32.375 | 7 |
| A0R2C4 | 1 | Uncharacterized protein | 1 | 1 | 0 | 0 | 0 | 0 | 1 | 1 | 0 | 0 | 0 | 9.2 | 16.473 | 0 | 7.2773 | 2 |
| A0R2D2 | 2 | Serine protease htrA | 1 | 2 | 0 | 0 | 2 | 0 | 0 | 0 | 0 | 0 | 0 | 5.2 | 51.631 | 0 | 11.995 | 2 |
| A0R2X5 | 1 | RDD family protein | 1 | 1 | 0 | 0 | 0 | 1 | 1 | 0 | 0 | 0 | 0 | 4.5 | 26.524 | 0 | 15.633 | 2 |
| A0R2Z1 | 2 | Hydrolase, CocE/NonD family protein | 1 | 2 | 0 | 0 | 0 | 0 | 0 | 2 | 0 | 0 | 0 | 5.5 | 62.865 | 0 | 14.82 | 2 |
| A0R3D0 | 1 | LpqT protein | 1 | 1 | 0 | 0 | 0 | 1 | 1 | 0 | 0 | 0 | 0 | 6.1 | 23.226 | 0 | 6.632 | 2 |
| A0R3I2 | 2 | Large conductance mechanosensitive channel protein | 1 | 2 | 0 | 0 | 0 | 2 | 2 | 0 | 0 | 0 | 0 | 21.8 | 16.626 | 0 | 42.805 | 8 |
| A0R3R8 | 2 | ABC transporter ATP-binding protein | 1 | 2 | 0 | 0 | 0 | 1 | 2 | 0 | 0 | 0 | 0 | 5.3 | 39.573 | 0 | 12.355 | 3 |
| A0R4C3 | 2 | Phosphate-binding protein PstS | 1 | 2 | 0 | 0 | 0 | 1 | 2 | 0 | 0 | 0 | 0 | 6.3 | 38.032 | 0 | 38.171 | 3 |
| A0R4G9 | 2 | Dyp-type peroxidase | 1 | 2 | 0 | 0 | 0 | 0 | 0 | 0 | 0 | 2 | 0 | 10.8 | 37.216 | 0 | 18.298 | 2 |
| A0R4H0 | 4 | 29 kDa antigen CFP29 | 1 | 4 | 0 | 1 | 0 | 0 | 0 | 0 | 2 | 4 | 0 | 15.8 | 28.73 | 0 | 42.349 | 14 |
| A0R582 | 2 | Probable conserved transmembrane protein rich in alanine | 1 | 2 | 0 | 0 | 0 | 0 | 0 | 2 | 1 | 0 | 0 | 4.5 | 55.679 | 0 | 12.869 | 4 |
| A0R588 | 4 | ATP-dependent zinc metalloprotease FtsH | 1 | 4 | 0 | 0 | 0 | 0 | 0 | 3 | 2 | 2 | 0 | 7.4 | 83.537 | 0 | 27.826 | 8 |
| A0R5D9 | 2 | DNA topoisomerase 1 | 1 | 2 | 0 | 0 | 0 | 0 | 0 | 2 | 0 | 0 | 0 | 3.3 | 102.5 | 0 | 12.111 | 2 |
| A0R5H5 | 1 | Anion-transporting ATPase | 1 | 1 | 0 | 0 | 0 | 1 | 1 | 0 | 0 | 0 | 0 | 3.5 | 36.835 | 0 | 6.4448 | 2 |
| A0R5K0 | 4 | ATPase family protein associated with various cellular activities (AAA) | 1 | 4 | 0 | 0 | 0 | 2 | 3 | 0 | 0 | 0 | 0 | 15.5 | 34.777 | 0 | 36.923 | 5 |
| A0R5Q4 | 1 | Integral membrane protein | 1 | 1 | 0 | 0 | 0 | 1 | 1 | 0 | 0 | 0 | 0 | 7.8 | 14.353 | 0 | 19.592 | 2 |
| A0R5T7 | 3 | Glutamine-binding periplasmic protein/glutamine transport system permease protein | 1 | 3 | 0 | 0 | 0 | 2 | 3 | 0 | 0 | 0 | 0 | 9.3 | 50.874 | 0 | 37.434 | 7 |
| A0R611 | 1 | Galactan 5-O-arabinofuranosyltransferase | 1 | 1 | 0 | 0 | 0 | 1 | 1 | 0 | 0 | 0 | 0 | 2.1 | 67.2 | 0 | 6.3128 | 3 |
| A0R616 | 2 | Propionyl-CoA carboxylase beta chain | 1 | 2 | 0 | 0 | 0 | 0 | 2 | 0 | 0 | 0 | 0 | 5.8 | 56.169 | 0 | 13.55 | 2 |
| A0R628 | 2 | Galactofuranosyltransferase GlfT2 | 1 | 2 | 0 | 0 | 1 | 0 | 1 | 1 | 0 | 0 | 0 | 3.6 | 71.572 | 0 | 11.817 | 3 |
| A0R696 | 2 | Glycine/D-amino acid oxidase | 1 | 2 | 0 | 0 | 0 | 0 | 1 | 2 | 1 | 0 | 0 | 4.6 | 52.004 | 0 | 12.849 | 4 |
| A0R6C7 | 2 | Uncharacterized protein | 1 | 2 | 0 | 0 | 0 | 2 | 1 | 0 | 0 | 0 | 0 | 15 | 15.482 | 0 | 18.947 | 3 |
| A0R6E9 | 3 | ABC Polyamine/Opine/Phosphonate transporter | 1 | 3 | 0 | 0 | 0 | 3 | 1 | 0 | 0 | 0 | 0 | 11.7 | 39.982 | 0 | 17.617 | 4 |
| A0R7F9 | 1 | 30S ribosomal protein S6 | 1 | 1 | 0 | 0 | 0 | 1 | 1 | 0 | 0 | 0 | 0 | 12.2 | 10.185 | 0 | 6.7067 | 4 |

**Supplementary Table S3. Mass spectrometry SDS-PAGE 40 kDa Band**

| **Score** | **Expectation** | **Protein ID** | **Protein Name** | **MW (kDa)** | **% Coverage** | **Comment** | **empai** |
| --- | --- | --- | --- | --- | --- | --- | --- |
| 119 | 8.3E-09 | A0QZY7 | LppL protein | 35.048 | 13.9 |  | 0.39 |
| 104 | 0.00000025 | A0QT42 | ABC transporter | 38.744 | 15.3 |  | 0.45 |
| 98 | 0.0000011 | A0R157 | Saccharopine dehydrogenase | 43.606 | 8.9 |  | 0.22 |
| 86 | 0.000016 | A0R4G9 | Dyp-type peroxidase | 37.193 | 6.4 |  | 0.26 |
| 82 | 0.000044 | A0R5H5 | Anion-transporting ATPase | 36.813 | 9.9 |  | 0.26 |
| 82 | 0.000046 | A0QTI0 | L-seryl-tRNA(Sec) selenium transferase | 43.941 | 4.9 |  | 0.14 |
| 65 | 0.002 | A0QUM6 | Hydrogenase-2, small subunit | 35.147 | 3.4 | Tentative 1 significant peptide | 0.09 |
| 58 | 0.011 | A0QSL8 | DNA-directed RNA polymerase subunit alpha | 37.896 | 3.4 | Tentative 1 significant peptide | 0.08 |
| 54 | 0.025 | A0R3S7 | Non-homologous end joining protein Ku | 35.69 | 8.5 |  | 0.27 |
| 54 | 0.028 | A0QRS0 | ABC-type transport system periplasmic substrate-binding protein | 44.507 | 5.1 |  | 0.14 |
| 49 | 0.076 | A0QQF8 | Glycosyl hydrolase family protein 76 | 43.431 | 2.8 | Tentative 1 significant peptide | 0.07 |
| 48 | 0.11 | A0QX32 | Band 7 protein | 43.956 | 5.1 |  | 0.14 |
| 42 | 0.39 | A0QU53 | Putative acyl-CoA dehydrogenase | 43.481 | 3.7 |  | 0.14 |
| 39 | 0.92 | A0QR89 | Geranylgeranyl reductase | 43.068 | 1.8 | Tentative 1 significant peptide | 0.07 |
| 36 | 1.7 | A0QTV6 | Monooxygenase | 42.711 | 2.6 | Tentative 1 significant peptide | 0.07 |

**Supplementary Table S4. Mass spectrometry SDS-PAGE 46 kDa Band**

| **Score** | **Expectation** | **Protein ID** | **Protein Name** | **MW (kDa)** | **% Coverage** | **Comment** | **empai** |
| --- | --- | --- | --- | --- | --- | --- | --- |
| 964 | 2.9E-93 | A0QPE7 | Oxidoreductase, short chain dehydrogenase/reductase family protein | 46.47 | 40 |  | 2.47 |
| 766 | 1.7E-73 | A0R5H6 | Ion-transporting ATPase | 40.314 | 37.5 |  | 2.9 |
| 298 | 9.7E-27 | A0QQF8 | Glycosyl hydrolase family protein 76 | 43.431 | 16.8 |  | 0.49 |
| 78 | 0.000098 | A0QR89 | Geranylgeranyl reductase | 43.068 | 3.8 |  | 0.14 |
| 69 | 0.0008 | A0QPE8 | 3-ketoacyl-CoA thiolase | 45.205 | 6 |  | 0.21 |
| 58 | 0.011 | A0R4G8 | Probable M18 family aminopeptidase 2 | 44.851 | 2.4 | Tentative 1 significant peptide | 0.07 |
| 20 | 65 | A0R408 | Sensor-type histidine kinase PrrB | 47.789 | 1.6 | Tentative 1 significant peptide | 0.06 |
